# Effects of physical, virtual reality-based, and brain exercise on physical, cognition, and preference in older persons: a randomized controlled trial

**DOI:** 10.1186/s11556-018-0199-5

**Published:** 2018-10-02

**Authors:** Thwe Zar Chi Htut, Vimonwan Hiengkaew, Chutima Jalayondeja, Mantana Vongsirinavarat

**Affiliations:** Faculty of Physical Therapy, Mahidol University, 999 Phutthamonthon 4 Rd., Salaya, Phutthamonthon, Nakhon Pathom, 73170 Thailand

**Keywords:** Balance, Cognition, Exercise, Exertion, Fall, Muscle strength, Older persons, Physical activity

## Abstract

**Background:**

Physical exercise (PE), virtual reality-based exercise (VRE), and brain exercise (BE) can influence physical and cognitive conditions in older persons. However, it is not known which of the three types of exercises provide the best effects on physical and cognitive status, and which exercise is preferred by older persons. This study compared the effects of PE, VRE, and BE on balance, muscle strength, cognition, and fall concern. In addition, exercise effort perception and contentment in older persons was evaluated.

**Methods:**

Eighty-four older persons (*n* = 84) were randomly selected for PE, VRE, BE, and control groups. The exercise groups received 8-week training, whereas the control group did not. Balance was assessed by Berg Balance Scale (BBS) and Timed Up and Go test (TUG), muscle strength by 5 Times Sit to Stand (5TSTS) and left and right hand grip strength (HGS), cognition by Montreal Cognitive Assessment (MoCA) and Timed Up and Go test Cognition (TUG-cog), fall concern by Fall Efficacy Scale International (FES-I), exercise effort perception by Borg category ratio scale (Borg CR-10), and exercise contentment by a questionnaire.

**Results:**

After exercise, PE significantly enhanced TUG and 5TSTS to a greater extent than VRE (TUG; *p* = 0.004, 5TSTS; *p* = 0.027) and BE (TUG; *p* = 0,012, 5TSTS; *p* < 0.001). VRE significantly improved MoCA (*p* < 0.001) and FES-I (*p* = 0.036) compared to PE, and 5TSTS (*p* < 0.001) and FES-I (*p* = 0.011) were improved relative to BE. MoCA was significantly enhanced by BE compared to PE (*p* < 0.001) and both MoCA and TUG-cog were improved compared to VRE (*p* = 0.04). PE and VRE significantly (*p* < 0.001) increased Borg CR-10 in all exercise sessions, whereas BE showed a significant improvement (*p* < 0.001) in the first 4 sessions. Participants had a significantly greater satisfaction with BE than controls (*p* = 0.006), and enjoyed VRE and BE more than PE (*p* < 0.001). Subjects in all exercise groups exhibited benefits compared to the control group (*p* < 0.001).

**Conclusions:**

PE provided the best results in physical tests, VRE produced measurable improvements in physical and cognition scores, while BE enhanced cognition ability in older persons. Older persons preferred VRE and BE compared to PE. Both exercises are suggested to older persons to improve physical and cognitive conditions.

## Background

Older persons often exhibit impairment in balance, muscle strength, cognition, and physical activity which can limit normal functions [1] and may lead to fall [2]. The use of several different exercises such as aerobic exercise, balance exercise, resistance exercise, exergames, and complex sports like martial arts can enhance physical function and cognition [3–17]. However, not all exercises are suitable to every older persons.

Physical exercise (PE) is conventional exercise such as aerobic, resistance, flexibility, and balance exercises that are often recommended for older persons [18]. PE can improve not only balance and physical function [3–7], but also cognition [8–14] in older persons. Aerobic exercise [19–22] and resistance exercise [11, 23], both separately and in combination [9, 20], can improve cognition in older persons. Additionally, aerobic and balance exercises link to executive function, a component of cognition [24]. Likewise, coordination exercise can improve cognition in older persons [25–27], appearing to involve in perceptual speed and visual-spatial network [25, 26]. Aerobic and coordination exercises are concluded to be more beneficial to cognitive process than stretching and balance exercises [13, 14]. One of the best ways to improve cognition with PE is to combine several kinds of PE [14]. Although PE is a standard exercise and is important to cognition in older persons, the exercise may carry risk for them due to age-related physiological deteriorations such as osteoporosis and sarcopenia [28–30]. Vertebral fractures in performing yoga spinal flexion positions [31], and shoulder injuries during progressive resistance training [32] were reported in older persons. These conditions make PE an inappropriate choice for many older persons that require an exercise program. Moreover, review studies propose less effective in cognitive improvement in pure PE alone than combining PE with cognitive training [12–14]. The multi-domain training is therefore suggested for older persons [12].

Virtual reality-based exercise (VRE) involves both physical and mental exercises and requires players to respond to sensory input to perform various tasks in technological stimulated scenarios [17]. VRE, such as playing virtual reality games, could be an alternative to PE for exercise therapy since VRE improves motor, and cognitive abilities [16, 17, 33–36] as well as muscle strength and balance in older persons [15, 16]. However, meta-analytic studies in action video game training [34, 35] and computerized cognitive training [36] demonstrate small benefit in both overall cognition and specific cognitive domains in older persons. Older persons benefit less from action video game training than healthy young adults in cognition [34] and show ineffective for executive function and verbal memory from computerized cognitive training [36]. Thus, suggestion of VRE to older persons and its effect on cognition in older persons still need investigations, particularly comparison with PE. Furthermore, some older persons may be unacquainted with VRE technology. This exercise technique may not be suitable for all people.

Brain exercise (BE) is an exercise technique that can improve cognitive function [37] allowing older persons to better perform basic daily activities [38]. The BE technique can be either technology based [39, 40] or utilize traditional games and activities [41]. Playing brain stimulating video games improves executive function and processing speed [39, 40], as well as short and long term memory [40]. In addition, playing board games promotes interest, planning, and memory, as well as reducing depression and anxiety in older persons [41]. Declines in cognitive abilities lead to difficulty in performing basic activities required for daily living [42–45], and increase the chance of injury inducing falls [46–48] in older persons. Thus, brain exercise promoting cognition [40] is assumed to influence balance and physical abilities in older persons who cannot use either PE or VRE techniques.

Currently, the benefit of PE, VRE, and BE on different mental and physical parameters remains unclear with evidence describing which exercise is superior for enhancement of physical or cognitive ability lacking. In addition, which of these exercise techniques are preferred by older persons has not been reported. Therefore, the goals of this study were to 1) compare effects of PE, VRE, and BE on balance, muscle strength, cognition, and fall concern in older persons, and 2) determine exercise effort perception and exercise contentment in older persons.

## Methods

### Participants and design

The study was a single-blind (investigator blinded but not participants) randomized controlled trial with within and between group comparisons. Participants were recruited from 2 homes for the aged in Yangon, informed about the study, and screened for study inclusion and exclusion. Inclusion criteria were as follows: 65–85 years, normal cognition by having a Mini-Mental State Examination (MMSE) score greater than 23 [49, 50], Barthel Index score of 100 [51], and passing medical screening by a physician prior to study entry. Exclusion criteria were as follows: any obvious symptom of diseases (e.g., heart disease, diabetes mellitus, hypertension), any neurological disorders (e.g., stroke, parkinsonism), visual, vestibular, and auditory impairments, active arthritis, joint arthroplasty or fusion, any limb amputation or surgery, and psychological problems.

**Fig. 1:**
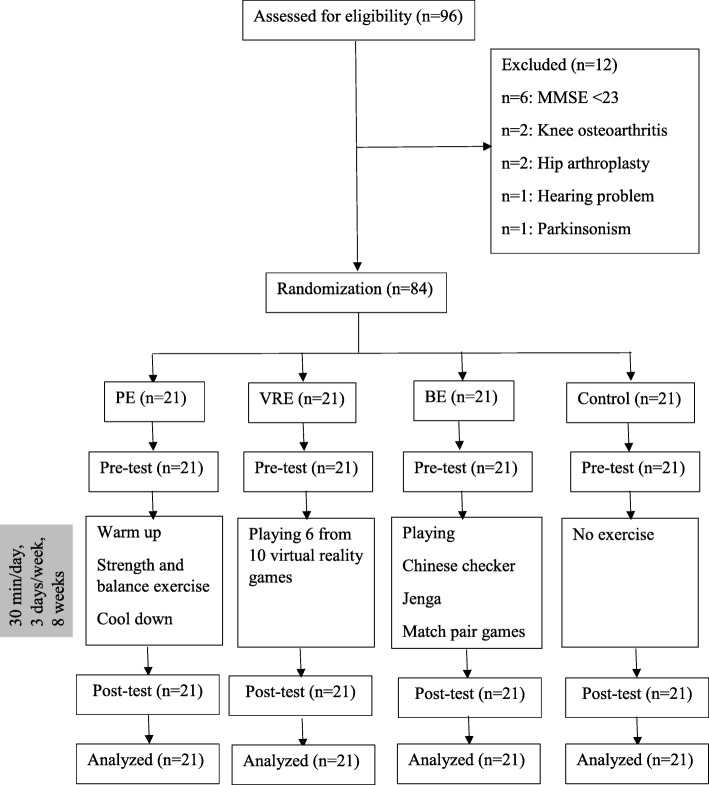
Randomization of participants into physical exercise (PE), virtual reality-based exercise (VRE), brain exercise (BE), and control group

Ninety-six (*N* = 96) older persons were qualified for the study, 12 of them were excluded (Fig. 1). Therefore, eighty-four (*N* = 84) older persons were in the study. They were randomly allocated by lottery with age matching within a range of 5 years into 4 groups; PE (*n* = 21), VRE (*n* = 21), BE (*n* = 21) and control (*n* = 21) (Fig. 1). Participants in the 4 groups were similar in age, body mass index, cognition, and education years. Characteristics of all participants and subgroups are shown in Table 1.

**Table 1:** Characteristics of all participants and participants in each subgroup: physical exercise (PE), virtual reality-based exercise (VRE), brain exercise (BE), and control. Data presenting in mean ± standard deviation (minimum-maximum) or n (%)

Characteristics	All (n = 84)	PE (n = 21)	VRE (n = 21)	BE (n = 21)	Control (n = 21)	*p*-value
Age (years)	75.8 ± 5.19	75.9 ± 5.65	75.8 ± 4.89	75.6 ± 5.33	76.0 ± 5.22	0.996
	(66–85)	(66–85)	(67–83)	(66–85)	(67–85)	
Gender						
Male	47 (56%)	13 (62%)	10 (48%)	12 (57%)	12 (57%)	–
Female	37 (44%)	8 (38%)	11 (52%)	9 (43%)	9 (43%)	–
Body mass Index (kg/m^2^)	23.3 ± 29.9	23.7 ± 4.24	22.6 ± 2.45	24.4 ± 2.32	22.7 ± 2.47	0.161
	(17.4–31.6)	(17.4–31.6)	(17.9–28.0)	(19.4–28.7)	(18.7–27.0)	
MMSE (scores)	25.2 ± 1.17	24.7 ± 0.96	25.5 ± 1.22	25.2 ± 1.41	25.2 ± 1.00	0.156
	(24–28)	(24–27)	(24–28)	(24–28)	(24–27)	
Education (years)	9.15 ± 2.95	8.81 ± 2.46	8.67 ± 2.27	9.62 ± 3.44	9.52 ± 3.53	0.643
	(5–15)	(6–15)	(5–15)	(5–15)	(5–15)	
Education level						
Graduate	11 (13%)	4 (19%)	2 (9.5%)	1 (5%)	4 (19%)	–
High school	33 (39%)	8 (38%)	7 (33%)	9 (43%)	9 (43%)	–
Middle school	31 (37%)	7 (33%)	12 (57%)	9 (43%)	3 (14%)	–
Primary school	9 (11%)	2 (9.5%)	0 (0%)	2 (9%)	5 (24%)	–

*MMSE* Mini Mental State Examination

### Procedure

The study was conducted at homes for the aged in which participants lived. At the homes for the aged participants had the same daily timetable such as having breakfast, lunch, dinner, praying, and free time. Cooperation of staff of homes for the aged, a physiotherapist arranged a certain schedule for participants in the PE, VRE, and BE groups that participants did together at the free time. A physiotherapist conducted exercises in the study. Participants in the PE, VRE, and BE groups received strength and balance exercise, virtual reality games, and brain games, respectively. The exercises took 30 min per day, 3 non-consecutive days per week for 8 weeks. Participants in the control group did not receive any exercise, they conducted their lives as usual. All participants recorded their activities of daily living and the logs were confirmed by staff of home for the aged.

Before starting the assessments, a researcher demonstrated the tests until participants understood, and allowed them to practice one trial. All participants did the assessments before and after 8 weeks of exercise. They were assessed for balance by Myanmar-translated Berg Balance Scale (BBS) permitted by Berg K [52] and Timed Up and Go test (TUG) [53], muscle strength by 5 Times Sit to Stand (5TSTS) [54] and hand grip strength (HGS) of the left and right hands [55], cognition by Myanmar-translated Montreal Cognitive Assessment (MoCA) permitted by Nasreddine ZS [56] and Timed Up and Go test Cognition (TUG-cog) [57], and fall concern by Myanmar-translated Fall Efficacy Scale International (FES-I) permitted by Yardley L [58]. In addition, participants evaluated perception of exercise effort using the Borg category ratio scale (Borg CR-10) [59] before and after every session of exercise. Moreover, they evaluated exercise contentment using a questionnaire after 8 weeks of exercise.

### Exercises

#### Physical exercise (PE)

The 30-min PE consisted of 5-min for warm-up and cool-down, and 20-min of strength and balance exercises. The warm-up consisted of elbow curls, hamstring stretch, quadriceps stretch, gastrosoleus stretch, and marching in place. The strength and balance exercises were chest press, horizontal pull, sit to stand squat, seated hip abduction, heel and toes raise, single leg stance, tandem stance, sideway walk, and tandem walk. The cool-down involved elbow circles, elbow curls, hamstring stretch, quadriceps stretch, and ankle circles. After 4 weeks of exercise, a physiotherapist evaluated changes in strength and balance in individual participants, and progressed the exercises by increasing intensity and/or resistance using an elastic band (TheraBand®), or decreasing support such as support from hands.

#### Virtual reality-based exercise (VRE)

Ten games from X-box 360 (Flextronics, Wistron, Celestica, Foxconn) were chosen: 1) Light Raise (stepping forward, backward, or sideward), 2) Virtual Smash (moving upper and lower limbs with slightly bending trunk to crush the box on the left, right, and front), 3) Stack’ em Up (shifting weight and slightly bending trunk to drop boxes on the left or right space), 4) One Ball Roll (using the left and right hands reciprocally to throw a ball to knock over bottles), 5) Pin Push (using the left and right hands alternately to throw a ball to knock a bottle in various directions), 6) Super Saver (side-stepping to prevent the ball hit to the goal), 7) Target Kick (kicking a ball to hit the targets), 8) Play Paddle Panic (using one or both hands to catch and strike a ball), 9) Body Bally (using hands, feet, and head to strike a ball), and 10) Bamp Bash (stepping forward, backward, sideward with moving trunk to avoid objects thrown by the opponents). In 30 min of playing, participants chose 6 games involving upper and lower limb movements, and balance training. Participants progressed in game play when they obtained the highest score in a level of the game, and the game allowed the players to advance.

#### Brain exercise (BE)

The 30 min of BE consisted of Chinese checkers, Jenga, and Match Pair games. Each game was played for 10 min. In the first 4 weeks, 2 participants played together in each game, whereas in the last 4 weeks, 4 participants played together. In the first week, a physiotherapist matched participants randomly to play together. The physiotherapist subsequently matched participants obtaining similar scores in the previous week of game play.

Chinese checkers, Jenga, and Match pair games are board and card games to exercise the mind [60]. Chinese checkers is a strategy board game that a player completes all of his/her pieces across the hexagram-shaped board into the corner of the star opposite his/her starting corner using single-step moves or jumping over other pieces. The winner is the first person to finish all pieces into home or absolutely blocking the opponent’s way. Jenga is a physical and mental skill game that players take turns removing one block at a time from at any level of a tower constructed of 54 wooden blocks, and then place the block removed on the topmost of the tower. The winner is the last person to successfully remove and place a block. Match pairs is a memory game where player need to match pairs of cards, which all of the cards are laid face down on a table and 2 cards are flipped face up over each turn. The winner is the person with the most pairs.

### Outcome measures

#### Balance

##### Berg balance scale (BBS)

The BBS [52] is a functional balance measurement with a high reliability in older persons [61]. It consists of 14 items using a 5-point scale ranging from 0 to 4, with 0 representing the lowest point of function and 4 demonstrating the highest point of function. The range of the total score is from 0 to 56. Higher the scores indicate better balance ability in participants.

##### Timed up and go test (TUG)

The TUG [53] is a balance and mobility assessment with good test-retest reliability in community-dwelling older persons [58]. The time to complete rising from a chair, walking 3 m, turning around, walking back to the chair, and sitting down was counted in seconds by a stopwatch (Professional stopwatch, Model No. JS-519; Shenzhen Junsd Industry Co, Ltd.). The time of 12 s or less is considered normal TUG performance for community-dwelling older women [62], and the time of 8.39 s has been reported for community dwelling healthy older persons [63].

#### Muscle strength

##### Five times sit to stand (5TSTS)

The 5TSTS [54] is a functional lower limb muscle strength measurement with excellent reliability [64]. It counts the time to complete standing up and sitting down as quickly as possible 5 times. The acceptable time of performance is 11.4 s or less for 60–69 years, 12.6 s or less for 70–79 years, and 14.8 s or less for 80–89 years [65].

##### Hand grip strength (HGS)

The HGS can reflect overall muscle strength [66] and is measured by a handheld dynamometer (Takei Scientific Instrument Co., Ltd., Japan. T.K.K 5401). The measurement demonstrates excellent validity and reliability [67, 68]. Participants sit on a chair without armrests, flex their elbow at 90° in neutral position with arm beside the trunk, slightly extend the wrist, and hold the dynamometer in their hand [67]. Participants performed a maximum effort for 3 s. The HGS was done with both left and right hands. The unit for hand grip measurement is in kilograms.

#### Cognition

##### Montreal cognitive assessment (MoCA)

The MoCA [56] assesses cognitive function consisting of attention and concentration, executive functions, memory, language, visuoconstructional skills, conceptual thinking, calculations, and orientation. The assessment has excellent reliability and a score of 26 or above from a total 30 is determined normal [69].

##### Timed up and go test cognition (TUG-cog)

The TUG-cog [57] is an assessment evaluating function combining motor and cognitive tasks and has good reliability in older persons [63]. Participants counted backward in 3 from a randomly selected number between 20 and 100 while standing up from a chair, walking 3 m, turning around, walking back to the chair, and sitting down [63]. The time to complete the task counted in seconds using a stopwatch (Professional stopwatch, Model No. JS-519; Shenzhen Junsd Industry Co, Ltd.). The average time to perform TUG-cog is 9.82 s for community dwelling healthy older persons [63].

#### Fall concern

##### Fall efficacy scale international (FES-I)

The FES-I [58] is a 16-item questionnaire assessing fear of falling with values from 1 to 4 for each item. Participants answered the questionnaire independently. The score can range from 16 to 64 points with lower scores indicating less fear of falling. The assessment has good reliability [58, 70].

#### Exercise effort perception

##### Borg category ratio scale (Borg CR-10)

The Borg CR-10 [59] is a 10-point perceived exertion measurement with 0 reflecting nothing at all and 10 indicating maximum exertion. Participants performed the Borg CR-10 evaluation independently.

#### Exercise contentment

A 6-scale questionnaire was created to evaluate satisfaction, pleasure, and benefit of the exercise by the participants. The scale ranges from 1 to 6, where 1 was the least and 6 was the most. The questions are “How satisfied are you with the exercise?”, “How happy are you with the exercise?”, and “How advantageous do you find the exercise?” Participants answered the questions independently.

#### Data analysis

Data were analyzed using SPSS 18.0 (IBM, IL, USA). Kolmogorov–Smirnov test was used to examine the normality of the data distribution. The data was normal distributed. One-way ANOVA was used to assess age, body mass index, MMSE scores, and education years among the PE, VRE, BE, and control groups. Two-way mixed ANOVA with Bonferroni corrections was conducted to analyze main effect of group (PE, VRE, BE, and control) and time (before and after exercise), and its interaction on BBS, TUG, 5TSTS, left and right HGS, MoCA, TUG-cog, FES-I, and Borg-CR 10. Effect size of the significant effects was determined by Cohen’s d. Kruskal-Wallis and Mann-Whitney test was used to determine exercise contentment among the PE, VRE, BE and control groups. Statistical significance for all tests was accepted below the level of 0.05.

## Results

### Balance

#### BBS

**Fig. 2:**
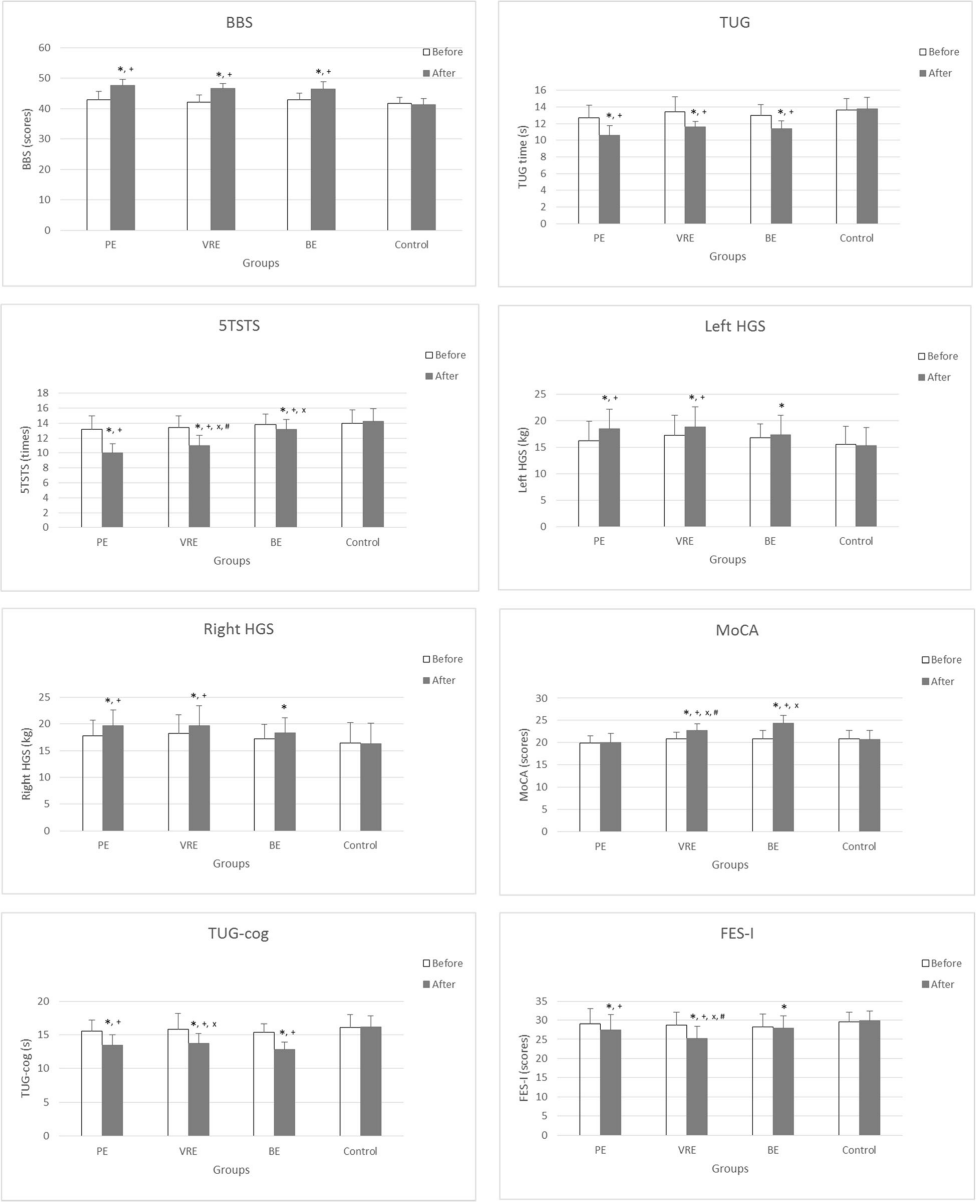
Berg balance scale (BBS), timed up and go test (TUG), five times sit-to-stand (5TSTS), left and right hand grip strength (HGS), timed up and go test cognition (TUG-cog), Montreal cognitive assessment (MoCA), and Fall efficacy scale international (FES-I) in physical exercise (PE), virtual reality-based exercise (VRE), brain exercise (BE), and control groups, ^*^ *p* < 0.05 significant difference from pre-test, ^+^ *p* < 0.05 significant difference from control at post-test, ^x^ *p* <0.05 significant difference from PE at post-test, ^#^ *p* < 0.05 significant difference from BE at post-test

There were significant main effects of groups (F_3,80_ = 12.7, *p* < 0.001), time (F_1,80_ = 446.8, *p* < 0.001), and its interaction (F_3,80_ = 67.3, *p* < 0.001) on BBS scores. Post hoc test revealed that BBS scores were significantly (*p* < 0.001) greater after PE, VRE, and BE than before exercise, with the effect size of PE = 1.29, VRE = 1.47, and BE = 1.21. The BBS scores after exercise of the three groups were significantly (*P* < 0.001) greater than the controls (Fig. 2) with the effect size of PE = 1.59, VRE = 1.65, and BE = 1.52.

#### TUG

There were significant main effects of groups (F_3,80_ = 10.6, *p* < 0.001), time (F_1,80_ = 162.6, *p* < 0.001), and its interaction (F_3,80_ = 25.5, *p* < 0.001) on TUG performance. Post hoc showed that TUG performance time after exercise was significantly (*p* < 0.001) reduced in PE, VRE, and BE compared with before exercise, with the effect size of PE = 1.26, VRE = 1.13, and BE = 1.14. After exercise, PE, VRE, and BE groups exhibited a significant (*P* < 0.001) decrease in TUG performance time compared with the controls, with the effect size of PE = 1.57, VRE = 1.43, and BE = 1.41. The PE group had a significant decrease in time compared with VRE (*p* = 0.004, effect size = 0.93) and BE (*p* = 0.012, effect size = 0.75) at post-test (Fig. 2).

### Muscle strength

#### 5TSTS

There were significant main effects of groups (F_3,80_ = 12.4, *p* < 0.001), time (F_1,80_ = 500.6, *p* < 0.001), and its interaction effect (F_3,80_ = 149.5, *p* < 0.001) on 5TSTS performance. The PE, VRE, and BE groups showed significant (*p* < 0.001) decrease in performance time after 8-week exercise, with the effect size of PE = 1.44, VRE = 1.26, and BE = 0.38. The time of the three groups after exercise was significantly reduced (PE, *p* < 0.001; VRE, *p* < 0.001; and BE, *p* = 0.036) when compared with controls showing the effect size of PE, VRE, and BE of 1.62, 1.42, and 0.60, respectively. Significantly decreased time was also observed in PE when compared with VRE (*p* = 0.027, effect size = 1.54) and BE (*p* < 0.001, effect size = 1.57). In addition, VRE showed significantly (*p* < 0.001, effect size = 1.27) less time than BE at post-test (Fig. 2).

#### HGS

For the left and right HGS, there were significant main effects of time (Left, F_1,80_ = 343.5, *p* < 0.001; Right, F_1,80_ = 514.7, *p* < 0.001) and interaction effect between groups and time (Left, F_3,80_ = 86.7, *p* < 0.001; Right, F_3,80_ = 86.2, *p* < 0.001). No main effect of groups (Left, F_3,80_ = 2.41, *p* = 0.073; Right, F_3,80_ = 2.68, *p* = 0.052) was observed. A significant (*p* < 0.001) increase in the left and right HGS was shown in PE (effect size, left = 0.62, right = 0.62), VRE (effect size, left = 0.40, right = 0.40), and BE (effect size, left = 0.23, right = 0.32) after exercise. In addition, HGS was significantly greater in PE (Left, *p* < 0.005; Right, *p* < 0.005) and VRE (Left, *p* < 0.005; Right, *p* < 0.005) than the controls, with the effect size for PE being 0.84 and 0.90, and for VRE was 0.88 and 0.83 for the left and right HGS, respectively. (Fig. 2).

### Cognition

#### MoCA

There were significant main effects of groups (F_3,80_ = 8.92, *p* < 0.001), time (F_1,80_ = 492.0, *p* < 0.001), and its interaction effect (F_3,80_ = 189.3, *p* < 0.001) on MoCA scores. Pairwise comparison showed that after exercise a significant (*p* < 0.001) increase in MoCA scores was shown in VRE (effect size = 1.08) and BE (effect size = 1.39). The scores of VRE and BE groups were significantly (*p* < 0.001) greater than those of PE (effect size: VRE = 1.32, BE = 1.56) and controls (effect size: VRE = 0.99, BE = 1.37). A significant (*p* = 0.04) decrease in MoCA score was observed in VRE (effect size = 0.88) when compared with BE (Fig. 2).

#### TUG-cog

There were significant main effects of groups (F_3,80_ = 6.48, *p* < 0.001), time (F_1,80_ = 308.1, *p* < 0.001), and its interaction effect (F_3,80_ = 40.7, *p* < 0.001) on TUG-cog performance. After exercise a significant (*p* < 0.001) decrease in TUG-cog time was shown in PE (effect size = 1.05), VRE (effect size = 0.95), and BE (effect size = 1.48). All exercises significantly (*p* < 0.001) decreased the time when compared with control (effect size: PE = 1.30, VRE = 1.25, BE = 1.53). Significantly (*p* = 0.04) increased time in TUG-cog was shown in VRE (effect size = 0.68) when compared with BE (Fig. 2).

### Fall concern

#### FES-I

There were significant main effects of time (F_1,80_ = 275.1, *p* < 0.001) and interaction effect between groups and time (F_3,80_ = 103.9, *p* < 0.001) on FES-I scores. No main effect of groups (F_3,80_ = 2.33, *p* = 0.081) was observed. Post hoc tests showed a significant decrease in FES-I scores in PE (*p* < 0.001, effect size = 0.43), VRE (*p* < 0.001, effect size = 0.97), and BE (*p* = 0.041, effect size = 0.10) after exercise. Furthermore, the scores were significantly lower in PE (*p* = 0.023, effect size = 0.67) than controls. The scores of the VRE group was significantly lower than PE (*p* = 0.036, effect size = 0.58), BE (*p* = 0.011, effect size = 0.77), and controls (*p* < 0.001, effect size = 1.24), as shown in Fig. 2.

### Perception of exercise effort

**Fig. 3:**
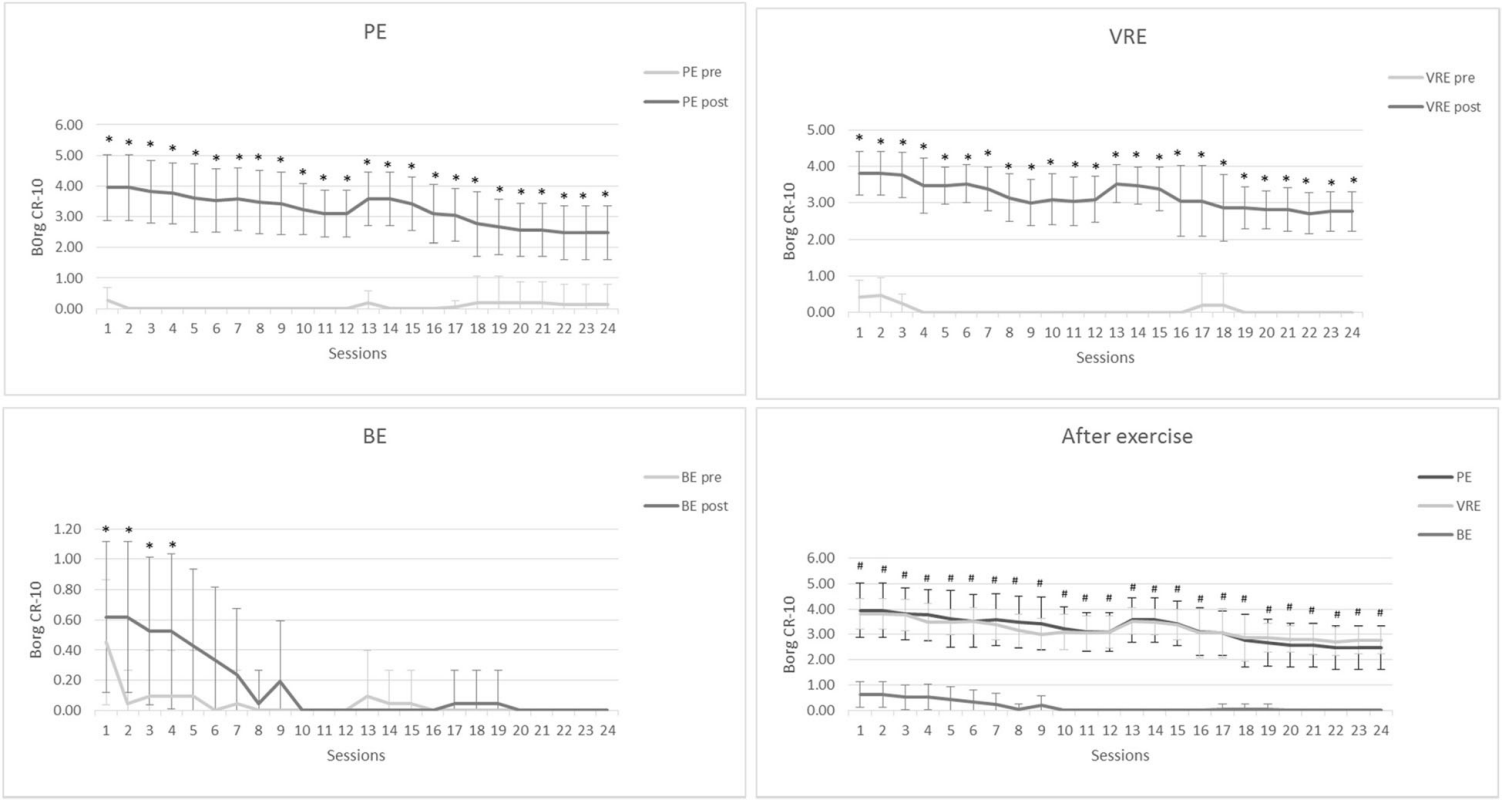
Perception of exercise effort measuring by Borg category ratio scale (Borg CR-10) in the group of physical exercise (PE), virtual reality-based exercise (VRE), and brain exercise (BE) at pre and post intervention, ^*^ *p* < 0.05 significant difference from pre-test, ^#^ *p* <0.05 significant difference in PE and VRE from BE

As shown in Fig. 3, after exercise the Borg CR-10 was significantly (*p* < 0.001) increased in all sessions of PE and VRE, but only in the first 4 sessions of BE. Furthermore, PE and VRE showed a significant (*p* < 0.001) higher Borg CR-10 than BE in all sessions.

### Exercise contentment

Among PE, VRE, BE, and control, there was a significant difference in exercise satisfaction (*p* = 0.046), pleasure (*p* < 0.001), and benefit (*p* < 0.001), as shown in Table 2. The BE group reported a significantly (*p* = 0.006) higher level of satisfaction than the control. Higher levels of pleasure were significantly (*p* < 0.001) shown in VRE and BE when compared with PE and control. Significantly (*p* < 0.001) higher benefit was shown in PE, VRE, and BE than in the control group.

**Table 2:** Satisfaction, pleasure, and benefit in physical exercise (PE), virtual reality-based exercise (VRE), brain exercise (BE), and control groups. Data presenting in mean ± standard deviation, and median (minimum-maximum)

	PE (n = 21)	VRE (n = 21)	BE (n = 21)	Control (n = 21)
Satisfaction	5.29 ± 0.72 5 (4–6)	5.33 ± 0.73 5 (3–6)	5.48 ± 0.51 ^+^ 5 (5–6)	4.90 ± 0.70 5 (3–6)
Pleasure	4.43 ± 0.51 4 (4–5)	5.76 ± 0.44 ^+, x^ 6 (5–6)	5.67 ± 0.66 ^+, x^ 6 (4–6)	3.90 ± 0.62 4 (3–5)
Benefit	5.10 ± 0.77 ^+^ 5 (4–6)	4.86 ± 0.85 ^+^ 5 (3–6)	5.38 ± 0.67 ^+^ 5 (4–6)	3.62 ± 0.80 4 (2–5)

^+^ *p* < 0.05 significant difference from control

^X^ *p* < 0.05 significant difference from PE

## Discussion

The purpose of the present study was to compare the effects of PE, VRE, and BE on balance, muscle strength, cognition, and fall concern, and to determine exercise effort perception and exercise contentment in older persons. PE, VRE, and BE routines were advantageous to balance, muscle strength, cognition, and fall concern in older persons. PE was important for balance and muscle strength, VRE for muscle strength, cognition, and fall concern, and BE for cognition. Older persons perceived exercise effort in PE and VRE. They were satisfied in BE, found both VRE and BE pleasurable, and gained benefit from all exercises.

The present findings demonstrate enhancement in balance, muscle strength, cognition, and fall concern in older persons after PE, VRE, or BE. The BBS and TUG values improved after exercise, demonstrating balance correction. The effect of PE on balance was not surprising as several exercises were directed to improve balance and lower limb muscle strength, for example single leg stance, tandem stance, sit to stand squat, and heel-toes raise. In addition, PE encouraged balance by increasing muscle strength. Participants in the PE group demonstrated decreased 5TSTS performance time and increased left and right HGS. The present finding supports the effect of PE on balance, similar to previous reports [3, 4, 6, 7].

The VRE in the present study was designed to move upper and lower limbs as well as trunk for balance training. This exercise routine enhanced balance by triggering weight shifting, in addition to improvement in 5TSTS performance and left and right HGS. Moreover, VRE increased MoCA scores which may affect balance since cognition and postural balance are associated in older persons [71–73]. The present findings agrees with previous reports [15, 16] on the effect of VRE on balance.

In the study the BE also improved balance. The BE routine promotes cognition that may be associated with balance and muscle strength improvement. Cognitive function is related to balance performance [74] and muscle strength [75, 76] which was evident in patients with cognitive impairment and Alzheimer disease. In the present study, the average MMSE scores of all participants was around 25 with the range of 24–28 indicating normal cognition [50]. However, their average MoCA scores before exercise was about 20 with the range of 18–25, showing cognitive impairment [69]. Participants in the BE group had cognitive impairment presented by the average MoCA score at pre-test of 20.8 with the range of 18–24. Therefore, BE may improve balance through enhanced cognitive function.

Although PE did not change MoCA scores, it did improve TUG-cog performance. This may be from greater improvement in physical function, 5TSTS, BBS, and TUG, than cognition alone. The effect of PE on cognition may require prolonged therapy since PE increases cerebral blood flow [77], and reduces neuroinflammation and other risk factors of cognition decline [78]. Previous studies have shown memory and cognitive improvement using 12-week balance training in healthy adults [79] and 6-month multi-exercise in older persons with mild cognitive impairment [80]. In the present study, 8 weeks of PE may be insufficient to enhance cognition. In contrast, VRE and BE may directly improve cognition, producing the improved TUG-cog performance.

In the present study, recovery of balance and muscle strength may promote confidence in doing tasks leading to decreased FES-I. The VRE routine appeared to be the best training for improvement of fall concern. A possible explanation is that VRE requires both physical and cognitive practice, whereas PE involves mainly physical and BE engages primarily in cognition. Among the three exercises, the present study indicates that PE improved balance with increased muscle strength, VRE enhanced balance via raised muscle strength and cognition, whereas BE improved balance with recovered cognition.

Older persons perceived exercise exertion in all sessions of PE and VRE, whereas they experienced effort in only first 4 sessions of BE. They also reported greater action levels in PE and VRE than BE. The PE and VRE routines involved the use of several large muscles, whereas BE engaged brain operations and small muscles, particularly of the hands. The score of Borg CR-10 after PE and VRE was 3–4, demonstrating moderate exercise effort, whereas that of BE was less than 1, corresponding to very light exertion. The greater score in PE and VRE could be explained by the need for using numerous muscle groups, resulting in increased exertion [81].

Although older persons involved in either exercise or non-exercise regimens demonstrated almost similar level of satisfaction in their tasks, those in BE group showed important satisfaction. Among PE, VRE, and BE, older persons derived pleasure from VRE and BE. They considered both exercises to be recreational activities. Furthermore, the BE had the highest rating for satisfaction, pleasure, and benefit of exercise. This might be related to social interaction as participants played these games two players at the time. From the effects of VRE and BE, these exercise routines are suggested to be given regularly to older persons unable to perform PE. Participants in the control group did not receive any intervention and did their daily activities according to the program of homes for the aged where they lived. Therefore, the passive control group might not have motivation compared to the exercise groups receiving different types of training, contacting a physiotherapist, and interacting with technology or persons. Consequently, they were less joyful and perceived less benefit from their programs.

The present study did comparison with a passive control group, however, the causal conclusions about the efficacy of PE, VRE, and BE could be drawn by other important components integrated in research methods [82, 83]. For example, the study was preregistration, random allocation, and blinding [82, 83]. There were two measures used in each outcome construct [82, 83]. Number of sample size was calculated with power analysis 0.8, the number in each group was large enough to observe effects [82, 83]. Training tasks were not similar to the outcome measures [84]. Participants in VRE group had never ever experienced with action video games [83, 84]. Those in BE group had no experience in board games, except one participant had experience in Chinese checker when he was young. In all outcome measures there was no difference between pre- and post-test in the control group, and no baseline difference among PE, VRE, BE, and control groups, presenting the control group being an acceptable control for placebo effect [84], and an adequate baseline for transfer effects [85]. Effect size of differences between exercise groups and the control were large in all outcome measures, except 5TSTS in BE and FES-I in PE were medium [82]. In the present study the effectiveness of PE, VRE, and BE were, therefore, reliable.

### Limitations

Participants lived at homes for the aged and had similar activities of daily living, for example washing clothes, cleaning beds and cupboards, praying, watching television, and having tea break in the afternoon. The participants in the study were very homogenous. In addition, brain games were designed to be played by two players. Hence, the present findings and the design of playing brain games may not be directly applicable to older persons living at home. Future study conducted in community-dwelling older persons is suggested to corroborate the present findings.

In the study there were several intervention groups. A task for the control group could not be designed to match to the exercise groups in term of task demands. The inappropriate task may lead the control group to be the inadequate active control condition [83, 84]. Thus, the control group was the passive control that was arranged in a real-world situation. To provide more powerful evidence for the effectiveness of interventions, particularly training with action video and broad games [83], future study would consider particular activities of the control group to the treatment group. Moreover, measurement of expectation on each outcome measure between the control and intervention groups is suggested [84, 85].

## Conclusions

PE, VRE, and BE promoted balance, muscle strength, cognition, and fall concern in independent daily living older persons. PE was the best for improving physical parameters, VRE enhanced both physical and cognition performance, while BE was effective in enhancing cognition. Older persons favored VRE and BE as these routines were considered more enjoyable. Thus, the present study suggests that VRE and BE can be used by older persons since both exercises directly improved physical performance and cognitive function that are a concern in older persons. In addition, our results suggest that VRE and BE may help fall concern in older persons.

## Abbreviations

5TSTS: 5 Times Sit to Stand
BBS: Berg Balance Scale
BE: Brain exercise
Borg CR-10: Borg category ratio scale
FES-I: Fall Efficacy Scale-International
HGS: Hand grip strength
MoCA: Montreal Cognitive Assessment
PE: Physical exercise
TUG: Timed Up and Go test
TUG-cog: Timed Up and Go test cognition
VRE: Virtual reality-based exercise
